# Heat Resistance Mediated by pLM58 Plasmid-Borne ClpL in *Listeria monocytogenes*

**DOI:** 10.1128/mSphere.00364-17

**Published:** 2017-11-01

**Authors:** Anna Pöntinen, Mariella Aalto-Araneda, Miia Lindström, Hannu Korkeala

**Affiliations:** Department of Food Hygiene and Environmental Health, Faculty of Veterinary Medicine, University of Helsinki, Helsinki, Finland; University of Iowa

**Keywords:** ClpL, *Listeria*, heat stress, heat tolerance, plasmid-mediated resistance, protease, stress response

## Abstract

*Listeria monocytogenes* is one of the most heat-resistant non-spore-forming food-borne pathogens and poses a notable risk to food safety, particularly when mild heat treatments are used in food processing and preparation. While general heat stress properties and response mechanisms of *L. monocytogenes* have been described, accessory mechanisms providing particular *L. monocytogenes* strains with the advantage of enhanced heat resistance are unknown. Here, we report plasmid-mediated heat resistance of *L. monocytogenes* for the first time. This resistance is mediated by the ATP-dependent protease ClpL. We tested the survival of two wild-type *L. monocytogenes* strains—both of serotype 1/2c, sequence type ST9, and high sequence identity—at high temperatures and compared their genome composition in order to identify genetic mechanisms involved in their heat survival phenotype. *L. monocytogenes* AT3E was more heat resistant (0.0 CFU/ml log_10_ reduction) than strain AL4E (1.4 CFU/ml log_10_ reduction) after heating at 55°C for 40 min. A prominent difference in the genome compositions of the two strains was a 58-kb plasmid (pLM58) harbored by the heat-resistant AT3E strain, suggesting plasmid-mediated heat resistance. Indeed, plasmid curing resulted in significantly decreased heat resistance (1.1 CFU/ml log_10_ reduction) at 55°C. pLM58 harbored a 2,115-bp open reading frame annotated as an ATP-dependent protease (ClpL)-encoding *clpL* gene. Introducing the *clpL* gene into a natively heat-sensitive *L. monocytogenes* strain (1.2 CFU/ml log_10_ reduction) significantly increased the heat resistance of the recipient strain (0.4 CFU/ml log_10_ reduction) at 55°C. Plasmid-borne ClpL is thus a potential predictor of elevated heat resistance in *L. monocytogenes*.

**IMPORTANCE** *Listeria monocytogenes* is a dangerous food pathogen causing the severe illness listeriosis that has a high mortality rate in immunocompromised individuals. Although destroyed by pasteurization, *L. monocytogenes* is among the most heat-resistant non-spore-forming bacteria. This poses a risk to food safety, as listeriosis is commonly associated with ready-to-eat foods that are consumed without thorough heating. However, *L. monocytogenes* strains differ in their ability to survive high temperatures, and comprehensive understanding of the genetic mechanisms underlying these differences is still limited. Whole-genome-sequence analysis and phenotypic characterization allowed us to identify a novel plasmid, designated pLM58, and a plasmid-borne ATP-dependent protease (ClpL), which mediated heat resistance in *L. monocytogenes*. As the first report on plasmid-mediated heat resistance in *L. monocytogenes*, our study sheds light on the accessory genetic mechanisms rendering certain *L. monocytogenes* strains particularly capable of surviving high temperatures—with plasmid-borne ClpL being a potential predictor of elevated heat resistance.

## Abstract (precis)

*Listeria monocytogenes* is a dangerous food pathogen causing the severe illness listeriosis that has a high mortality rate in immunocompromised individuals. Although destroyed by pasteurization, *L. monocytogenes* is among the most heat-resistant non-spore-forming bacteria. This poses a risk to food safety, as listeriosis is commonly associated with ready-to-eat foods that are consumed without thorough heating. However, *L. monocytogenes* strains differ in their ability to survive high temperatures, and comprehensive understanding of the genetic mechanisms underlying these differences is still limited. Whole-genome-sequence analysis and phenotypic characterization allowed us to identify a novel plasmid, designated pLM58, and a plasmid-borne ATP-dependent protease (ClpL), which mediated heat resistance in *L. monocytogenes*. As the first report on plasmid-mediated heat resistance in *L. monocytogenes*, our study sheds light on the accessory genetic mechanisms rendering certain *L. monocytogenes* strains particularly capable of surviving high temperatures—with plasmid-borne ClpL being a potential predictor of elevated heat resistance.

## INTRODUCTION

*Listeria monocytogenes* is a Gram-positive, non-spore-forming food-borne pathogen and the causative agent of listeriosis, a severe human illness with mortality reaching 35% (1–4). *L. monocytogenes* may persist in food-processing premises for several years (5, 6), which makes it a challenging contaminant in food production. In addition, it is able to cope with many stress conditions used for controlling bacterial contamination, including high temperatures (7, 8). *L. monocytogenes* can grow at 45°C and is more heat resistant than most other non-spore-forming food-borne pathogens (1, 9). Furthermore, a previous heat treatment may enhance the tolerance and adaptation of *L. monocytogenes* to subsequent heat stress as well as other stressors encountered in food production, such as NaCl and ethanol (7, 10). However, distinct differences exist between *L. monocytogenes* strains in their ability to survive high temperatures (10, 11). For example, Lundén et al. reported a 3-log-unit difference in the heat resistance (log_10_ reduction) of *L. monocytogenes* strains (11). While the general heat stress properties and adaptation responses of *L. monocytogenes* have been reported (8, 12), investigations are required to reveal the accessory genetic mechanisms that provide certain strains with enhanced heat resistance.

Among mobile genetic elements, plasmids are self-replicating entities that are often costly, yet they may contribute to diversified adaptation and resistance of the host strain (13, 14). Plasmids are relatively prevalent among *L. monocytogenes* strains: approximately one-third of *L. monocytogenes* strains harbor plasmids (15–17), and they are particularly found in environmental and food-related strains (15). Thus, their potential to contribute to the environmental fitness of the host cannot be overlooked. Indeed, the involvement of listerial plasmids in resistance of *L. monocytogenes* to antibiotics (18, 19), benzalkonium (20–22), and heavy metals (15, 23) has been reported. The role of plasmids is yet to be elucidated, however, in the adaptation of *L. monocytogenes* strains into niches of food production environments, where high temperature is a key stressor for bacteria to surmount.

Due to the severe risk on food safety posed by markedly heat-resistant *L. monocytogenes* strains, it is pivotal to better understand the variation in their ability to survive heat treatments. Here, we sought to elucidate the genetic mechanisms conferring heat resistance in *L. monocytogenes* by comparing the genome composition and heat survival phenotypes. We show that heat resistance is mediated by the plasmid-borne ATP-dependent protease ClpL. To the best of our knowledge, this is the first report on plasmid-mediated heat resistance in *L. monocytogenes*.

## RESULTS

### Resistance and growth of *L. monocytogenes* strains vary at high temperatures.

**FIG 1:**
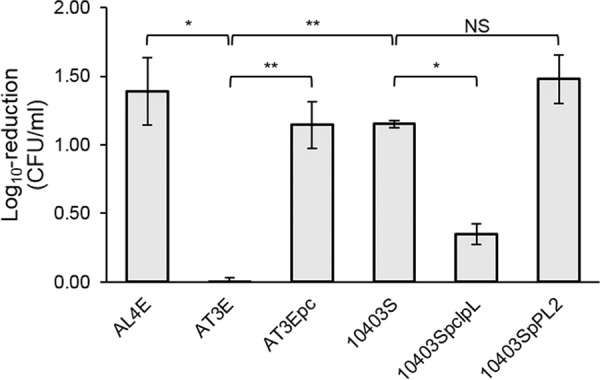
Susceptibility of *L. monocytogenes* strains to heat stress at 55°C after 40 min. Plasmid curing in the heat-resistant strain AT3E led to significantly impaired survival at 55°C. Introducing *clpL* into the heat-sensitive 10403S strain resulted in significantly increased heat resistance, while control vector pPL2 without *clpL* did not have the same effect. These findings suggest that pLM58 is involved in heat resistance of *L. monocytogenes* and that this resistance is mediated by the plasmid-borne ATP-dependent protease ClpL. The log_10_ reduction values are the means ± standard deviations (error bars) for three replicate cultures. Statistical significance was determined using the independent-samples two-tailed *t* test and indicated as follows: *, *P* < 0.01; **, *P* < 0.001; NS, not significant.

**FIG 2:**
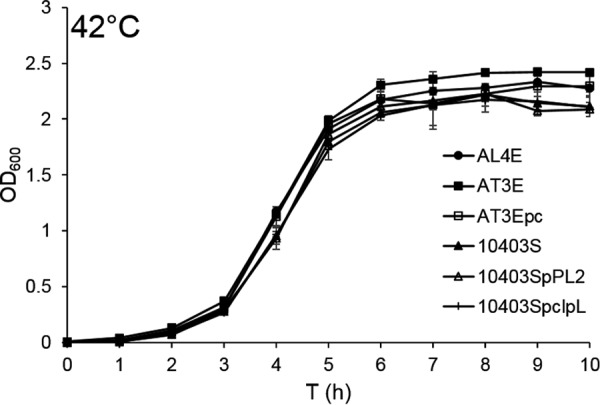
Growth of *L. monocytogenes* strains AL4E, AT3E, and 10403S, derivative cured strain AT3Epc, and conjugation strains 10403Sp*clpL* and 10403SpPL2 displayed no significant differences at 42°C. All strains were grown in BHI broth for 10 h, and the OD_600_ was measured every hour. Time (in hours) is shown on the *x* axis. The data represent the mean OD_600_ values ± standard deviations (error bars) for three replicate cultures. The correspondence between the OD_600_ values and viable-cell numbers was verified by plate counts at mid-logarithmic and stationary growth phase.

We first tested the heat resistance at 55°C, growth at 42°C, and maximum growth temperature of *L. monocytogenes* AL4E and AT3E (Table 1) in order to elucidate the differences between their thermoresistance and growth at high temperature. With 0.0 CFU/ml log_10_ reduction, *L. monocytogenes* AT3E proved to be more heat resistant than AL4E (1.4 CFU/ml log_10_ reduction; *P* < 0.01) at 55°C (Fig. 1). At 42°C, the differences between their growth were negligible (Fig. 2). Strain AL4E exhibited 0.5°C higher maximum growth temperature than the heat-resistant AT3E strain did (*P* < 0.01).

**TABLE 1:** Bacterial strains and plasmids used in this study

Strain or plasmid	Description or relevant phenotype or characteristic	Reference or source^a^
*L. monocytogenes*		
AL4E	Wild-type strain; serotype 1/2c	11
AT3E	Wild-type strain; serotype 1/2c	11
AT3Epc	AT3E strain; plasmid-cured strain	This study
10403S	Wild-type strain; serotype 1/2a; streptomycin resistant	27
10403Sp*clpL*	10403S with tRNA^Arg^::p*clpL*	This study
10403SpPL2	10403S with tRNA^Arg^::pPL2	This study

*E. coli*		
NEB5α	Chemically competent strain	New England Biolabs
HB101	Conjugation donor containing helper plasmid pRK24	CRBIP

Plasmids		
pLM58	Plasmid in the AT3E strain	This study
p*clpL*	pPL2 containing 423 bp of upstream nucleotides and coding sequence of ATP-dependent protease *clpL*	This study
pPL2	Site-specific integration vector	61

^a^ CRBIP, Biological Resource Centre of the Institut Pasteur.

### *L. monocytogenes* AL4E and AT3E share high chromosomal sequence identity.

**FIG 3:**
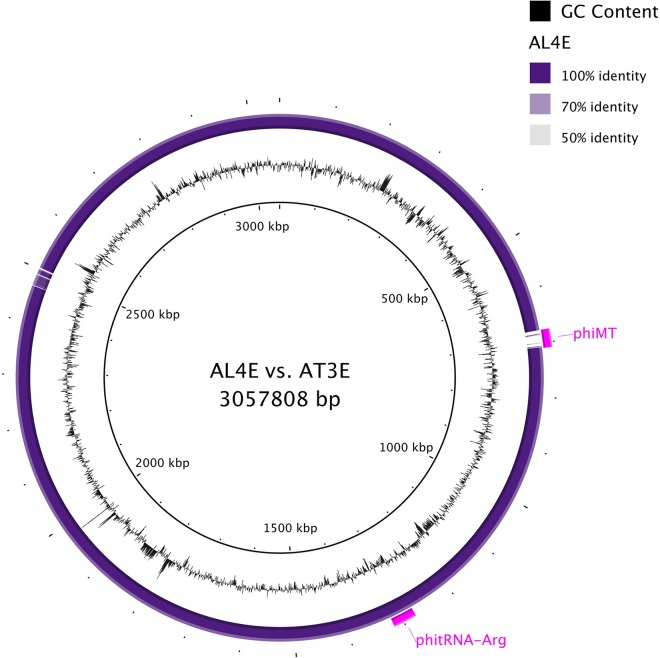
*L. monocytogenes* AL4E and AT3E strains share high chromosomal sequence identity as shown in the BLAST circular representation generated using BRIG (BLAST ring image generator). The middle circle shows GC content. The outer circle shows BLAST comparison of strains AL4E and AT3E, and sequence identity of AL4E to the chromosomal sequence of strain AT3E is coded by color as indicated in the color key in the figure. Intact phage inserts of AT3E are designated.

In order to identify genetic differences explaining the variation in heat resistance phenotypes between the strains, we compared the genome composition of the newly sequenced AL4E and AT3E strains. Both strains were of serotype 1/2c and multilocus sequence type (MLST) ST9 and had a GC content of 38% (Table 2). They shared high chromosomal identity (Fig. 3). Genome comparison of the strains in SEED Viewer 2.0 (24) revealed 49 chromosomal genes unique to strain AT3E and 21 chromosomal genes unique to strain AL4E; most of these genes were hypothetical or phage related. PHASTER (25) predicted two intact phages, of which the 42.7-kb phage insert (designated ϕtRNA-Arg) adjacent to the arginine tRNA gene was related to *Listeria* phage LP-101 and present in both strains. The 33.5-kb phage insert (designated ϕMT) downstream of a methyltransferase gene was related to *Listeria* phage A006 and absent in strain AL4E (Fig. 3).

**TABLE 2:** General features of *L. monocytogenes* strains and plasmid sequenced in this study

Strain or plasmid	MLST type^a^	Assembly size (bp)	GC content (%)	No. of predicted CDS^b^	No. of RNAs	Avg length (bp) of CDS	Origin	Country	Yr of isolation
AL4E	ST9	3,027,995	38.0	3,002	85	885	Equipment, conveyor	Finland	1998
AT3E	ST9	3,057,808	38.0	3,049	85	881	Product, sausage	Finland	1995

pLM58	NA	58,523	36.6	70	0	714	AT3E strain	Finland	2017

^a^ NA, not applicable.

^b^ CDS, coding sequences.

### *L. monocytogenes* AT3E harbors a novel 58-kb plasmid.

**FIG 4:**
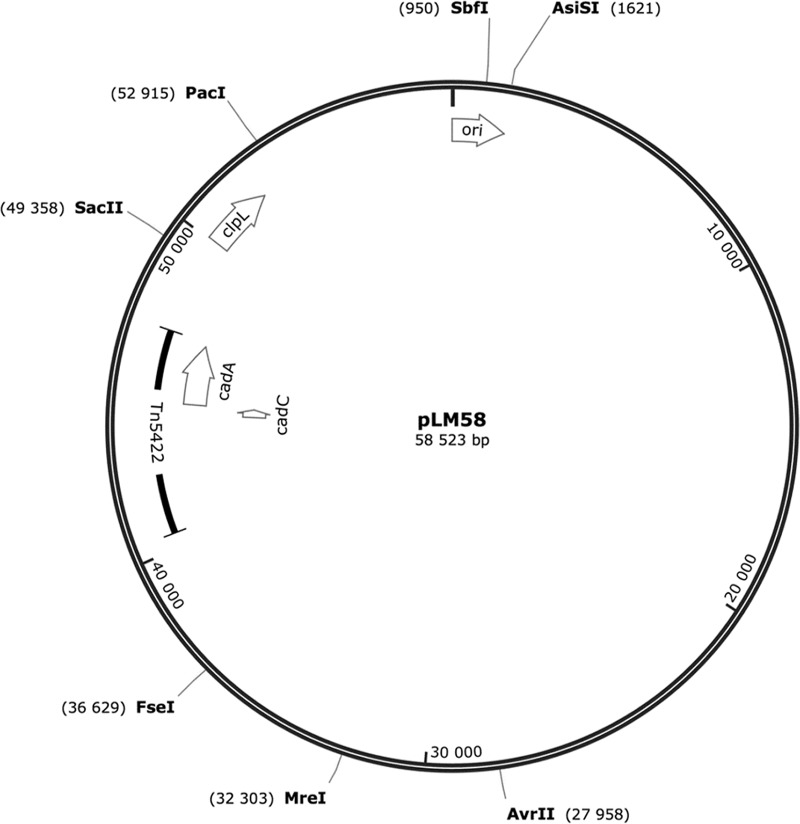
Genetic map of *L. monocytogenes* plasmid pLM58 built using SnapGene Viewer 3.3.4. Initiation of the replication protein-encoding *ori* gene, ATP-dependent protease-encoding *clpL* gene, cadmium resistance genes *cadA* and *cadC*, transposon Tn*5422*, and unique restriction sites is indicated.

Upon genome analysis, we discovered that the heat-resistant strain AT3E harbors a novel plasmid, which was designated pLM58. It is 58.5 kb in size and contains 70 predicted open reading frames (ORFs) and 19 predicted operons and has a GC content of 36.6% (Fig. 4 and Tables 2 and 3). In sequence comparison with *L. monocytogenes* plasmids deposited in GenBank at NCBI, pLM58 manifested a mosaic structure characteristic of listerial plasmids.

Annotation of pLM58 revealed an ORF that putatively encodes an ATP-dependent Clp protease ATP-binding subunit (ClpL) and is unrelated to any predicted operons (Fig. 4 and Table 3). An identical *clpL* sequence was harbored by 55% (12/22) of the different *L. monocytogenes* plasmids in GenBank. No *clpL* sequence was present in the remainder of the plasmids deposited in NCBI. Furthermore, *clpL* of pLM58 shared high nucleotide sequence identity (98%; E value of 0.0) with *clpL2* of *Lactobacillus rhamnosus* and moderate amino acid identity with plasmid-borne *clpK2* of *Escherichia coli* (46%; E value of 9E−175).

**TABLE 3:** Putative open reading frames and their functions in pLM58

ORF no.	Start position	Stop position	Length (bp)	Strand^a^	GC content (%)	Predicted operon^b^	Function (RAST annotation^c^)
1	1	1635	1,635	+	42.81		Replication initiation protein
2	1736	1900	165	+	38.79	Op1	Hypothetical protein
3	1910	2665	756	+	41.53	Op1	FIG00775790; hypothetical protein
4	3301	2699	603	−	35.49	Op2	Mobile element protein
5	4133	3378	756	−	37.04	Op2	Mobile element protein
6	5370	4144	1,227	−	36.51	Op2	Mobile element protein
7	5714	6304	591	+	29.78	Op3	FIG00775444; hypothetical protein
8	6307	7224	918	+	33.88	Op3	FIG00775381; hypothetical protein
9	7622	7861	240	+	37.50	Op4	Pli0011 protein
10	7873	8313	441	+	34.01	Op4	Pli0010 protein
11	8306	9646	1,341	+	36.99	Op4	FIG00776061; hypothetical protein
12	9657	10028	372	+	38.44	Op4	FIG00775078; hypothetical protein
13	10048	10812	765	+	41.05	Op4	Pli0008 protein
14	10926	11456	531	+	36.72		Pli0007 protein
15	11828	11493	336	−	35.52		Pli0006 protein
16	12002	12400	399	+	34.83	Op5	Pli0005 protein
17	12404	13906	1,503	+	38.92	Op5	DNA methylase
18	13991	14443	453	+	36.42	Op5	Mobile element protein
19	15152	14472	681	−	40.38	Op6	Mobile element protein
20	15584	15183	402	−	37.56	Op6	Transposase and inactivated derivative-like protein
21	15776	17140	1,365	+	40.44		NADH peroxidase (EC 1.11.1.1)
22	18255	17386	870	−	41.49		l-Proline glycine betaine ABC transport system permease protein ProW (TC 3.A.1.12.1)
23	18376	18615	240	+	32.92	Op7	Hypothetical protein
24	18615	18935	321	+	29.91	Op7	Pli0046 protein
25	19165	19890	726	+	39.94		FIG00774464; hypothetical protein
26	20353	21282	930	+	44.95	Op8	Lead-, cadmium-, zinc-, and mercury- transporting ATPase (EC 3.6.3.3) (EC 3.6.3.5); copper-translocating P-type ATPase (EC 3.6.3.4)
27	21270	22235	966	+	45.55	Op8	Lead-, cadmium-, zinc-, and mercury- transporting ATPase (EC 3.6.3.3) (EC 3.6.3.5); copper-translocating P-type ATPase (EC 3.6.3.4)
28	22972	22292	681	−	38.18		Mobile element protein
29	23785	23411	375	−	33.60		Hypothetical protein
30	24131	24006	126	−	31.75		Hypothetical protein
31	24307	24447	141	+	33.33	Op9	Hypothetical protein
32	24461	24829	369	+	44.17	Op9	DNA topoisomerase III (EC 5.99.1.2)
33	24822	25010	189	+	33.33	Op9	Hypothetical protein
34	25734	26156	423	+	44.92	Op10	Mannose-6-phosphate isomerase (EC 5.3.1.8)
35	26240	26893	654	+	39.60	Op10	ABC-type uncharacterized transport system; ATPase component
36	26890	27654	765	+	40.13	Op10	YbbM seven-transmembrane-helix protein
37	27839	28036	198	+	41.92		Mobile element protein
38	28362	28478	117	+	30.77		Hypothetical protein
39	28459	28578	120	+	28.33		Hypothetical protein
40	28571	29143	573	+	37.87		Nonspecific DNA-binding protein Dps/ iron-binding ferritin-like antioxidant protein/ferroxidase (EC 1.16.3.1)
41	29112	29273	162	+	30.25		FIG00630535; hypothetical protein
42	29288	29992	705	+	43.55	Op11	Transcriptional regulator; Crp/Fnr family
43	30085	30309	225	+	42.67	Op11	Prolipoprotein diacylglyceryl transferase (EC 2.4.99.-)
44	30456	30689	234	+	36.32		Copper chaperone
45	30887	32791	1,905	+	41.99		Copper-translocating P-type ATPase (EC 3.6.3.4)
46	35030	33174	1,857	−	29.35	Op12	Bipolar DNA helicase HerA
47	36216	35023	1,194	−	28.14	Op12	FIG036446; hypothetical protein
48	37063	36485	579	−	39.90		Resolvase/integrase bin
49	37324	39369	2,046	+	34.55	Op13	Lead-, cadmium-, zinc-, and mercury- transporting ATPase (EC 3.6.3.3) (EC 3.6.3.5); copper-translocating P-type ATPase (EC 3.6.3.4)
50	39384	40544	1,161	+	29.72	Op13	Multicopper oxidase
51	43467	40552	2,916	−	36.83	Op14	Mobile element protein
52	44025	43471	555	−	38.02	Op14	DNA invertase
53	44305	44664	360	+	35.28	Op15	Cadmium efflux system accessory protein
54	44664	46799	2,136	+	39.98	Op15	Cadmium-transporting ATPase (EC 3.6.3.3)
55	46867	47271	405	+	37.53	Op15	Multicopper oxidase
56	47291	47836	546	+	33.15	Op15	DUF1541 domain-containing protein
57	47988	48110	123	+	21.95		Hypothetical protein
58	48221	48820	600	+	38.33	Op16	Site-specific recombinase; resolvase family
59	48810	49037	228	+	35.96	Op16	Hypothetical protein
60	49435	49157	279	−	36.92		FIG00775972; hypothetical protein
61	49398	49634	237	+	34.60		Conserved hypothetical protein; phage associated
62	50062	52176	2,115	+	40.00		ATP-dependent protease ATP-binding subunit *clpL*
63	52443	53441	999	+	35.04	Op17	Mobile element protein
64	53457	53792	336	+	36.01	Op17	Mobile element protein
65	53946	54701	756	+	32.54		FIG00775080; hypothetical protein
66	54906	55250	345	+	41.16	Op18	FIG00775387; hypothetical protein
67	55238	56533	1,296	+	41.82	Op18	ImpB/MucB/SamB family protein
68	56832	56536	297	−	29.63	Op19	Hypothetical protein
69	57784	56810	975	−	30.46	Op19	Replication-associated protein
70	58142	58300	159	+	28.93		Hypothetical protein

^a^ The location of the open reading frame on the plus (+) or minus (−) strand is indicated.

^b^ Operon prediction was performed using FGENESB (54).

^c^ Annotations were performed using RAST 2.0 (52).

pLM58 did not carry any antibiotic resistance genes but harbored genes similar to *cadAC* that mediate cadmium resistance in *Staphylococcus aureus* (23) (Table 3). The *cadAC* genes were associated with a transposon Tn*5422*-related sequence (Fig. 4) and colocalized in an operon encoding predicted multicopper oxidase and a protein with unknown function (Table 3). The replication initiation protein of pLM58 shared high sequence identity (99%; E value of 0.0) with plasmids that have been allocated into group 1 of listerial plasmids by replicon-based distinction (26). Finally, no intact phages were found in pLM58.

### Plasmid pLM58 contributes to heat resistance of *L. monocytogenes* AT3E.

In order to confirm whether heat resistance could be mediated by pLM58, the heat-resistant strain AT3E was cured of plasmid and subjected to heat resistance and growth assays. Plasmid curing resulted in the removal of plasmid from 2% (1/56) of *L. monocytogenes* AT3E colonies. The cured derivative strain AT3Epc showed significantly impaired heat resistance compared to the AT3E parent, with a log_10_ reduction of 1.1 CFU/ml (*P* < 0.001) at 55°C (Fig. 1). The maximum growth temperatures and the kinetic growth parameters of the cured strain AT3Epc and its parent strain AT3E did not differ (*P* > 0.05).

### Plasmid-borne ATP-dependent protease gene *clpL* increases heat resistance in a natively heat-sensitive strain.

The *clpL* gene was introduced into heat-sensitive *L. monocytogenes* 10403S (Table 1) (27) in order to ascertain whether the plasmid-mediated heat-resistant phenotype was attributable to the ATP-dependent protease ClpL harbored by pLM58. Conjugation of *clpL* in the pPL2 backbone increased the heat resistance of the recipient strain 10403S, which was observed by a significant decrease in log_10_ reduction from 1.2 CFU/ml to 0.4 CFU/ml (*P* < 0.01) at 55°C (Fig. 1). Conjugation of the control plasmid pPL2 lacking *clpL* did not increase heat resistance of strain 10403S but did lead to a slight decrease in cell concentration (log_10_ reduction 1.5 CFU/ml), although the difference was not statistically significant (*P* > 0.05). No significant differences were observed in the maximum growth temperature or kinetic growth parameters at 42°C between strain 10403S and conjugation strain 10403Sp*clpL* or 10403SpPL2 (*P* > 0.05).

### pLM58 is putatively nonconjugative.

In order to confirm whether pLM58 is self-transmissible, standard plate mating was performed between strains AT3E and 10403S. The plasmid-borne *cadAC* genes confer cadmium resistance (17, 23) and were also found in pLM58 (Table 3). Thus, cadmium resistance facilitates the selection of recipient cells that have not received pLM58. The innate streptomycin resistance of strain 10403S facilitates the selection of possible transconjugants from the donor strain (28). The AT3E donor strain and 10403S recipient strain grew on positive-control plates containing 130 μg/ml CdSO_4_ or 200 μg/ml streptomycin, respectively. However, no colonies were detected on selective plates containing both 130 μg/ml CdSO_4_ and 200 μg/ml streptomycin. Colonies on selective plates containing less cadmium sulfate (65 μg/ml) tested positive by PCR for strain 10403S and negative for plasmid pLM58. Indeed, pLM58 also lacked the known type IV secretion system genes needed for the conjugation process of self-transmissible plasmids (29–31) (Table 3).

## DISCUSSION

For the purpose of elucidating the genetic mechanisms that render certain *L. monocytogenes* strains particularly resistant to heat, the genome sequences of a heat-resistant and heat-sensitive wild-type strain were compared. The two chromosomal sequences were highly similar. Yet, genome sequence analysis revealed a 58-kb plasmid exclusively harbored by the resistant AT3E strain, which suggested that the observed phenotypic difference in heat resistance between the two strains may be mediated by a plasmid. Indeed, plasmid curing resulted in significant reduction of cell concentration at 55°C, while the parent AT3E strain survived for the measured 40-min period. To the best of our knowledge, this is the first description of plasmid-mediated heat resistance in *L. monocytogenes*.

In comparison to previously reported listerial plasmids, heat resistance-mediating pLM58 is medium sized with 58 kb and 70 predicted ORFs (Tables 2 and 3). Listerial plasmids are mosaics of highly homologous fragments (32–34). Indeed, pLM58 is a novel plasmid harboring fragments both unique and highly similar to closely related plasmids. By replicon-based distinction of listerial plasmids, pLM58 was allocated into group 1 that manifests relatively small plasmid genomes (26).

Although plasmids have been shown to contribute to the resistance of *L. monocytogenes* to stressors such as antibiotics (18, 19), disinfectant (20–22), and heavy metals (15, 23), little evidence is available on thermal resistance attributable to listerial plasmids. Hingston et al. discovered by genetic characterization that the presence of plasmids is associated with cold sensitivity of *L. monocytogenes* (16). Studying *Listeria innocua* strains, Margolles and de los Reyes-Gavilán found no difference in thermal inactivation by pasteurization between the Li16 strain and its cured derivative Li16c (35). Therefore, they suggested that pLI59 harbored by strain Li16 does not encode genes related to heat resistance (35). Heat stress-related genes have been annotated in *L. monocytogenes* plasmids (36), but phenotypic evidence on their importance in growth or survival at high temperatures has been lacking thus far.

pLM58 harbored a 2,115-bp ORF annotated as *clpL* putatively encoding ATP-dependent protease ClpL that we considered a potential mediator of heat resistance in *L. monocytogenes*. Indeed, introducing the putative promoter and the coding sequence of *clpL* into the natively heat-sensitive *L. monocytogenes* 10403S innately lacking *clpL*, resulted in significantly increased survival at 55°C. Conjugation of the control vector pPL2 without *clpL* did not have the same effect, which indicates that the vector itself does not confer resistance to heat treatment. These findings suggest that plasmid-borne *clpL* plays a role in elevated heat resistance of *L. monocytogenes*. The presence of the same ORF in many other listerial plasmids suggests that heat resistance mediated by *clpL* may be widespread among *L. monocytogenes* strains harboring plasmids. Clp ATPases function both as ATP-dependent proteases degrading damaged and misfolded proteins and as chaperones involved in protein folding (37). The chromosomally encoded ClpC, ClpP, and ClpE class III heat shock proteins are involved in virulence and stress tolerance of *L. monocytogenes* (38, 39). However, our study is the first to describe heat resistance attributable to a plasmid-borne Clp in *L. monocytogenes*.

The* clpL* gene of pLM58 was nearly identical to the plasmid-borne *clpL* of *L. rhamnosus*. As the *clpL* homolog in *L. rhamnosus* is surrounded by transposase genes and was mobilizable (40), it has been proposed that the ClpL protease is acquired via horizontal gene transfer (33). Canchaya et al. also suggest that ClpL of *L. monocytogenes* may originate from lactic acid bacteria (33). Interestingly, *clpL* of pLM58 is surrounded by genes related to site-specific recombinases, phages, and other mobile genetic elements (Table 3), further evidence for the putative horizontal transfer of *clpL*. *clpL* expressed from an *L. rhamnosus* plasmid was upregulated during heat shock (40). It is thus possible that plasmid-borne *clpL* plays a universal role in heat resistance of Gram-positive bacteria.

While ClpL is exclusively associated with Gram-positive bacteria (41), we found that *clpL* of pLM58 is moderately similar to plasmid-borne *clpK* of the Gram-negative *E. coli*. Intriguingly, plasmid-mediated heat resistance has been reported in an *E. coli* dairy isolate (42) and in a nosocomial *Klebsiella pneumoniae* strain (41). In both studies, thermotolerance was shown to correlate with the presence of a plasmid-borne ATPase-encoding *clpK* gene (41, 42).

Plate mating between the AT3E donor strain and the 10403S recipient strain yielded no transconjugants, which suggests that pLM58 is not self-transmissible. This is in line with the fact that pLM58 did not harbor any known type IV secretion system genes needed for the conjugation process of self-transmissible plasmids (29–31). It remains to be verified whether pLM58 is mobilizable. However, listerial plasmids manifest mosaic patterns (33), and we found *clpL* among the ones reported in NCBI. Therefore, the heat resistance-mediating *clpL* gene could also be found in a conjugative plasmid. Therefore, the conjugative ability of listerial plasmids harboring *clpL* should be further investigated.

In addition to the presence of pLM58, the heat-resistant AT3E strain harbored an intact phage insert, related to *Listeria* phage A006 and absent in the heat-sensitive AL4E strain. Many phages encode virulence factors contributing to bacterial pathogenesis as well as stress resistance genes specifically related to survival of bacteria in host cells (43). However, further investigations are needed to elucidate their potential in conferring bacterial heat resistance.

Although proven heat sensitive at 55°C, strain AL4E had a slightly higher maximum growth temperature than the heat-resistant AT3E strain did. In addition, the differences between their growth at 42°C were negligible. Furthermore, plasmid curing in the heat-resistant AT3E strain or introducing the *clpL* gene into the heat-sensitive 10403S strain had no effect on maximum growth temperature or kinetic growth parameters at 42°C. This suggests that the mechanisms underlying resistance to thermal kill are different from those permitting growth at the higher end of the growth temperature range. Bojer et al. demonstrated that the maximum growth temperature of *K. pneumoniae* was unaffected by a mutation in *clpK* that was shown to mediate heat resistance in the bacterium (41). Studying growth and survival under acid stress, Metselaar et al. demonstrated that increased acid resistance in *L. monocytogenes* was, in fact, correlated with decreased maximum growth rate (44). Heterogeneity under different stress conditions may be of advantage to *L. monocytogenes*, since it may benefit cell survival (44).

In addition to increased environmental fitness of bacteria, plasmid-borne stress resistance genes are of concern due to potential cotransfer with virulence and antibiotic resistance genes often harbored by plasmids (36, 41, 45). Thus, they may enhance the ability of plasmid-harboring pathogens to survive in different niches, which creates opportunities to infect new hosts.

This study is, to the best of our knowledge, the first description of a plasmid that plays a role in heat resistance of *L. monocytogenes*. We state that plasmid-borne ATP-dependent protease ClpL contributes to the survival of *L. monocytogenes* at high temperature. Plasmid-borne ClpL is a potential predictor of elevated heat resistance in *L. monocytogenes* and other Gram-positive bacteria. Our findings bring light to accessory genetic mechanisms that cause large variation in the ability of *L. monocytogenes* strains to survive heat treatments.

## MATERIALS AND METHODS

### Strains and growth conditions.

The bacterial strains and plasmids used are presented in Table 1. The strains were routinely grown at 37°C on blood agar, on tryptic soy agar (TSA) or in tryptic soy broth (TSB), on brain heart infusion (BHI) agar or in BHI broth, on Luria-Bertani (LB) agar or in LB broth (Oxoid, Cheshire, England), or on ALOA (Harlequin Listeria Chromogenic) agar (Labema, Helsinki, Finland). Appropriate antibiotics (Sigma-Aldrich, St. Louis, MO) and cadmium sulfate (3CdSO_4_ ⋅ 8H_2_O) (Merck, Darmstadt, Germany) were added when needed. *L. monocytogenes* AL4E and AT3E were serotyped according to the supplier’s instructions using the Listeria Antisera Set (Denka Seiken, Tokyo, Japan) including O- and H-factor antisera and by using the Pasteur typing tool (http://bigsdb.pasteur.fr/perl/bigsdb/bigsdb.pl?db=pubmlst_listeria_seqdef_public) (accessed 13 July 2017).

### Heat resistance assay.

The wild-type strains 10403S, AL4E, and AT3E, cured derivative strain AT3Epc, and conjugation strains 10403SpPL2 and 10403Sp*clpL* were challenged in a heat survival test conducted by the method of Lundén et al. (11) with minor modifications. Briefly, three colonies of each strain were individually inoculated into TSB and grown overnight at 37°C, followed by dilution (1:100) of each culture into 10 ml of fresh TSB and subsequent overnight incubation at 37°C to reach a cell concentration of 10^8^ to 10^9^ CFU/ml. The cultures were then diluted (1:100) into 5 ml of fresh TSB and placed in a 55°C water bath for 40 min. Serial dilutions from room temperature before heat stress (*t*0) and after 40 min at 55°C were plated on PCA agar (Oxoid) to determine the decrease in cell concentration (log_10_ reduction). Colony counting on PCA plates was performed after 2 days of incubation at 37°C. Strains showing a log_10_ reduction of less than 1.0 log_10 _were considered heat resistant.

### Growth curve analyses.

Three colonies of each *L. monocytogenes* strain were individually inoculated into 10 ml of BHI broth and incubated overnight at 37°C. The cultures were diluted (1:100) in fresh BHI broth and grown at 42°C for 10 h under continuous shaking, and the optical density at 600 nm (OD_600_) was measured at 1-h intervals. Growth parameters, including the maximum growth rate, lag phase, maximum optical density, and area under the curve were obtained for each strain using the grofit package (46) with default settings in R 3.2.2 (R Core Team, Vienna, Austria). The parameters were determined using the logistic model, which fitted the data best based on the Akaike information criterion (47). Correspondence between the OD_600_ values and viable-cell numbers of the strains was confirmed by plate counts at mid-logarithmic and stationary growth phases.

### Differences in maximum growth temperatures.

Differences in maximum growth temperatures of *L. monocytogenes* strains were examined using Gradiplate W10 incubator (BCDE Group, Helsinki, Finland) by the method of Hinderink et al. (48) with slight modifications. Briefly, three overnight cultures of each strain were individually diluted (1:100) in peptone water and plated on TSA (containing 25 g/liter agar) by the stamping technique in duplicate. The strains were grown for 24 h at a temperature gradient of 39.2°C to 45.7°C. Growth boundaries were determined by using a stereomicroscope (Olympus SZ61; Nikon, Tokyo, Japan).

### Genome sequencing and comparative genomic analysis.

Genomic DNA from *L. monocytogenes* AL4E and AT3E was extracted using guanidium thiocyanate by the method of Pitcher et al. (49). Whole-genome sequencing was performed by the Institute of Biotechnology (Helsinki, Finland) using single-molecule real-time (SMRT) sequencing in the PacBio RS platform with coverages of 199× (AL4E), 252× (AT3E), and 422× (pLM58). The genomes were *de novo* assembled using the RS_HGAP_Assembly.3 protocol (Pacific Biosciences of California, Inc., Menlo Park, CA). Multilocus sequence types (MLSTs) of strains AL4E and AT3E were derived using the Pasteur MLST typing tool (50, 51). The genomes were annotated using RAST 2.0 (52), and genome comparison was performed in SEED Viewer 2.0 (24). Prophage prediction was performed using PHASTER (25, 53), and sequence-based operon prediction of pLM58 was performed using FGENESB (54). Visual comparison of AL4E and AT3E chromosomes was generated using BRIG (BLAST ring image generator) (55), and pLM58 was visualized using SnapGene Viewer 3.3.4 (GSL Biotech LLC, Chicago, IL). Using BLASTN 2.2.26 (56), coding sequences of the replication initiation protein and the ATP-dependent protease ClpL harbored by pLM58 were compared to each *L. monocytogenes* plasmid sequence deposited in GenBank at NCBI (https://www.ncbi.nlm.nih.gov/genome/plasmids/159?; accessed 13 July 2017).

### Plasmid curing.

Plasmid curing in the heat-resistant AT3E strain was performed by the method of Margolles and de los Reyes-Gavilán (35). Briefly, strain AT3E was grown in TSB overnight at 37°C and inoculated (1:100) into TSB supplemented with a subinhibitory concentration of novobiocin (0.2 µg/ml) as a nonmutagenic curing agent (57, 58). The cultures were incubated at 40°C for 24 h, followed by nine identical subsequent inoculations (1:100) and incubations. The final cultures were plated on TSA plates containing novobiocin (0.2 µg/ml). The absence of the plasmid was verified from purified colonies by PCR using primers *oriV*-F (F stands for forward) and *oriV*-R (R stands for reverse) specific for the replication initiation protein-encoding gene of pLM58 and primers *clpL*-F and *clpL*-R specific for the ATP-dependent protease ClpL-encoding *clpL* gene harbored by pLM58 (Table 4). All the primers were designed using Primer3 (v. 4.0.0) (59, 60).

**TABLE 4:** Primers used in this study

Primer	Sequence (5′ − 3′)^a^	Reference
*oriV*-F	GAACAAGCGATCCGTCATGC	This study
*oriV*-R	TCGTTGCTAGGACTTGTCTGG	This study
*clpL*-F	ACAGGCTCGTGATGGCTTAC	This study
*clpL*-R	ACCGCGATATTGAGTTCCCG	This study
BamHI *clpL* FN	NNNNGGATCCAGTTTCAAAGGTCGTTCTGGC	This study
BamHI *clpL* RN	NNNNGGATCCTCTATCAAGCAATCTCCTTCCC	This study
NC16	GTCAAAACATACGCTCTTATC	61
PL95	ACATAATCAGTCCAAAGTAGATGC	61
ESAT-6 F	GCAATCAGTGGGAAGGACTG	This study
ESAT-6 R	ATCCATCGCTTGTTTTCCTG	This study
*secA*-F	ACTACTGCCAAAACATCGAAGC	This study
*secA*-R	AAGACGCACTGGATTCCCTC	This study

^a^ The bases in the sequences are shown as follows: N, any of the bases, i.e., adenine (A), cytosine (C), guanine (G), or thymine (T). Restriction sites in the sequences are underlined.

### Introducing *clpL* into heat-sensitive *L. monocytogenes*.

The coding sequence of *clpL* and a 423-bp upstream region, including the putative promoter, were conjugated into the heat-sensitive *L. monocytogenes* 10403S. This was done in order to verify the role of *clpL* in enhancing the heat resistance of *L. monocytogenes*. Conjugation was performed using the PSA prophage site-specific phage integration vector pPL2 (kindly provided by Martin Loessner, Swiss Federal Institute of Technology, Zurich, Switzerland) by the methods of Lauer et al. and Ma et al. (61, 62). Briefly, the coding sequence and the putative promoter of *clpL* were amplified by PCR using primers BamHI *clpL* FN and BamHI *clpL* RN (Table 4). The insert was ligated into the BamHI restriction site of linearized pPL2 treated with recircularization-preventing Antarctic phosphatase (New England Biolabs, Ipswich, MA) and propagated in *E. coli* NEBα (New England Biolabs). The p*clpL* and control plasmid pPL2 without an insert were separately transformed into the conjugation donor *E. coli* HB101 and conjugated into the 10403S recipient strain by filter mating. Transconjugants were selected on ALOA agar supplemented with 7.5 μg/ml chloramphenicol. Integration of the plasmids was confirmed using primers NC16 and PL95 (61), and the presence of the insert was confirmed using *clpL* gene-specific primers (Table 4).

### Horizontal transfer experiments.

To examine whether pLM58 is self-transmissible between two wild-type *L. monocytogenes* strains, standard plate mating was performed with AT3E as the donor strain and *L. monocytogenes* 10403S (kindly provided by Martin Wiedmann, Cornell University, Ithaca, NY) as the recipient strain. Briefly, the donor strain was grown in BHI, and the recipient strain was grown in BHI supplemented with 200 μg/ml streptomycin. After overnight growth at 37°C, 10403S cells were washed twice with fresh BHI, and both strain cultures were diluted (1:100) into fresh BHI and grown to logarithmic growth phase (OD_600 _of 0.5). Equal volumes (100 μl) of the donor and recipient were spotted on top of each other on BHI agar and incubated at room temperature for 1 h followed by 24-h incubation at 37°C. The cells were washed from the plate with BHI broth, and possible transconjugants were screened after 3 days of incubation at 37°C on BHI plates containing 200 μg/ml streptomycin and 65 μg/ml or 130 μg/ml CdSO_4_. The experiment was done in two simultaneous repeats, and the visible colonies were screened by PCR using primers specific for pLM58 *oriV*, ESAT-6 in the AT3E genome, and *secA* in the 10403S genome (Table 4). To serve as both positive and negative controls, the donor AT3E and recipient 10403S strains were individually plated on BHI agar containing 130 μg/ml CdSO_4_ or 200 μg/ml streptomycin.

### Statistical analysis.

Statistical analysis was conducted in IBM SPSS statistics 24 (IBM, Armonk, NY). Differences in log_10_ reductions at 55°C, growth parameters at 42°C, and maximum growth temperatures between the strains were tested using independent-samples two-tailed *t* test.

### Accession number(s).

The nucleotide sequences have been deposited in the GenBank database under accession numbers CP023754 (strain AL4E), CP023752 (strain AT3E), and CP023753 (plasmid pLM58) within BioProject PRJNA412588.
